# Tortuosidade das Artérias Coronárias como um Novo Fenótipo para Isquemia sem Doença Arterial Coronariana

**DOI:** 10.36660/abc.20210787

**Published:** 2022-09-15

**Authors:** André Estrada, André Silveira Sousa, Claudio Tinoco Mesquita, Humberto Villacorta

**Affiliations:** 1 Universidade Federal Fluminense Hospital Universitário Antônio Pedro Niterói RJ Brasil Universidade Federal Fluminense Hospital Universitário Antônio Pedro , Niterói , RJ – Brasil; 2 Hospital Pró-Cardíaco Rio de Janeiro RJ Brasil Hospital Pró-Cardíaco , Rio de Janeiro , RJ – Brasil; 3 Universidade Federal Fluminense Faculdade de Medicina Departamento de Radiologia Niterói RJ Brasil Universidade Federal Fluminense Faculdade de Medicina – Departamento de Radiologia , Niterói , RJ – Brasil; 4 Universidade Federal Fluminense Niterói RJ Brasil Universidade Federal Fluminense – Cardiologia, Niterói , RJ – Brasil

**Keywords:** Vasos Coronários, Isquemia, Isquemia Miocárdica

## Abstract

**Fundamento:**

As artérias coronárias tendem a ser mais tortuosas que outras artérias e acompanham os movimentos repetidos de flexão e relaxamento que ocorrem durante o ciclo cardíaco. A Tortuosidade das artérias Coronárias (TCor) causa alterações no fluxo coronariano, com uma redução na pressão de perfusão distal, o que pode levar à isquemia miocárdica.

**Objetivo:**

Avaliar a associação entre TCor e isquemia miocárdica.

**Métodos:**

Entre janeiro de 2015 e dezembro de 2017, 57 pacientes com angina e doença arterial coronariana não obstrutiva pela angiografia coronária invasiva (ACI) foram incluídos retrospectivamente. Variáveis angiográficas foram analisadas para avaliar a presença e grau de tortuosidade e correlacionadas com seus respectivos territórios vasculares na cintilografia de perfusão miocárdica com estresse. A TCor foi definida como artérias coronárias com três ou mais curvaturas com ângulos ≤ 90o, medidos durante diástole. Um nível de 5% foi estabelecido como estatisticamente significativo. Um nível de 5% foi definido como estatisticamente significativo.

**Resultados:**

Um total de 17 homens e 40 mulheres foram incluídos (idade média de 58,3 anos). A TCor foi observada em 16 pacientes (28%) e em 24 das 171 artérias. Observou-se uma associação significativa entre TCor e isquemia na análise por artéria (p<0,0001). O fator angiográfico mais associado com isquemia foi o número de curvaturas em uma artéria epicárdica medido na sístole (p=0,021).

**Conclusão:**

Este estudo mostrou uma associação da TCor com isquemia miocárdica em pacientes com artérias coronárias não obstruídas e angina. Observou-se uma relação entre número aumentado de curvaturas na artéria coronária medido por angiografia durante sístole e isquemia.

## Introdução

A doença cardíaca isquêmica é a principal causa de morte nos países desenvolvidos, e limita a qualidade de vida dos pacientes nos âmbitos físico, social, financeiro, e de saúde. ^1^ As recentes diretrizes da *European Society of Cardiology* para o diagnóstico e manejo das síndromes coronarianas crônicas (SCCs) descrevem cenários clínicos de pacientes com suspeita ou diagnóstico confirmado de SCC. ^2^ O perfil clínico de angina sem obstrução coronária tem sido cada vez mais reconhecido e associado com obesidade, intolerância à glicose, e expectativa de vida mais longa. ^3^ Estudos sugerem que até 55% dos pacientes encaminhados para angiografia coronária, mesmo com sintomas típicos, não apresentam obstruções; e até 40% dos pacientes com artérias normais ou quase normais (sem lesões obstrutivas) na angiografia coronária apresentam isquemia, como demonstrado nos testes de estresse. ^4^ Pacientes com angina pectoris que não apresentam obstrução coronária importante ainda têm risco aumentado para eventos cardiovasculares maiores, tais como morte cardiovascular, infarto agudo do miocárdio, acidente vascular cerebral, e mortalidade por todas as causas. ^5^ Esses pacientes apresentam também maior risco de insuficiência cardíaca com fração de ejeção preservada. ^6^

Um possível mecanismo relacionado à isquemia na doença não obstrutiva é a tortuosidade das artérias coronárias (TCor). A redução da pressão de perfusão distal e do fluxo coronariano, levando ao surgimento de isquemia do miocárdio pode ser observado em alguns casos de TCor. Há duas causas para essa redução de pressão: fricção devido ao estresse de cisalhamento e efeito centrífugo no interior das curvas. ^7^ Essa associação tem sido pouco abordada na literatura. O objetivo primário deste estudo foi avaliar a correlação entre TCor e isquemia do miocárdio em pacientes sem obstrução coronariana, e o segundo objetivo foi verificar as características geométricas de cada vaso coronário que poderiam estar correlacionadas com isquemia.

## Materiais e métodos

### Seleção dos pacientes

Este foi um estudo retrospectivo conduzido em dois centros médicos. O estudo foi aprovado pelo comitê de ética institucional. Todos os pacientes assinaram um termo de consentimento antes de participarem no estudo.

Um total de 57 indivíduos foram incluídos na análise final. Selecionamos pacientes que se submeteram a testes provocativos e apresentaram alterações isquêmicas, e à angiografia coronária que não mostrou obstruções. Desses pacientes, 28 haviam se submetido a um teste de exercício positivo, mas não haviam se submetido à cintilografia do miocárdio, o qual foi então realizado prospectivamente. O intervalo máximo entre cintilografia do miocárdio e angiografia coronária foi de um ano, independentemente da ordem em que os exames foram realizados.

Foram incluídos pacientes com idade igual ou maior a 18 anos, com queixa clínica de angina pectoris, submetidos à angiografia coronária invasiva (ACI) que revelou ausência de lesões obstrutivas (uma lesão não obstrutiva foi definida como ausência de obstrução ou uma obstrução inferior a 30%). Foram excluídos pacientes com qualquer das seguintes condições: insuficiência cardíaca, hipertensão pulmonar, doença congênita, doença valvular cardíaca, revascularização prévia do miocárdio (cirúrgica ou percutânea), cardiomiopatia hipertrófica, miocardite, ponte miocárdica, anomalias congênitas de origem coronariana (distribuição e curso), fístulas arteriovenosas, e microfístula entre a artéria coronária e o ventrículo esquerdo, espasmo coronariano induzido por cateter, anemia (hemoglobina < 10 g/d/L), bloqueio de ramo esquerdo ou uso de marcapasso definitivo.

### Dados clínicos

Foram realizadas revisão de prontuários médicos e entrevista com os pacientes. A classe funcional de angina foi verificada de acordo com os critérios da *Canadian Cardiology Society* (CCS), ^2^ além de sintomas associados de dispneia, história de comorbidades tais como hipertensão, diabetes mellitus, dislipidemia, história de tabagismo e inatividade física, e exames complementares disponíveis.

### Angiografia coronária invasiva

**Figura 1:**
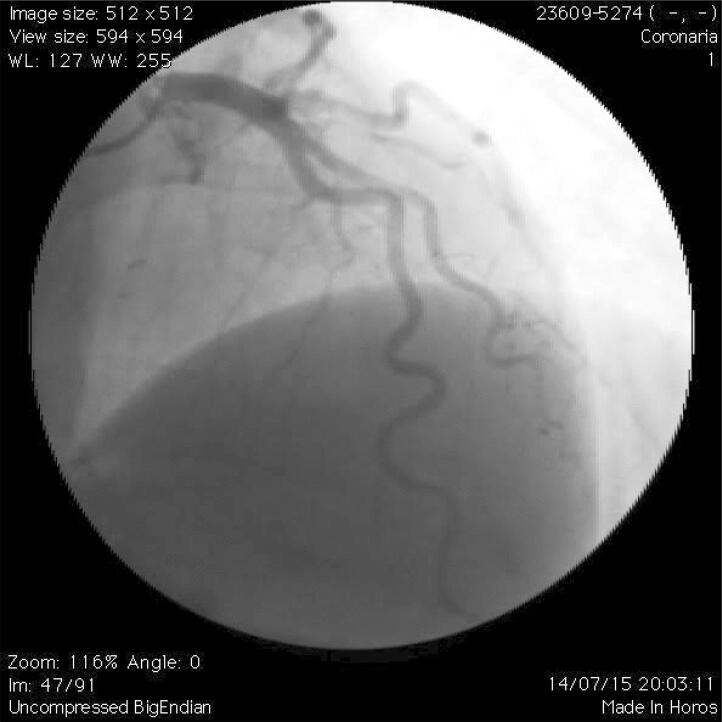
Angiografia coronária mostrando como é realizada a medida da curvatura (graus).

A ACI foi utilizada para excluir presença de obstruções coronárias e pontes miocárdicas, e avaliar a presença e o grau de TCor. Análise quantitativa da ACI foi realizada usando técnicas padronizadas. A TCor foi definida como a presença de pelo menos três curvaturas consecutivas, com um ângulo de curvatura inferior a 90 graus e de uma artéria coronária epicárdica maior que 2 mm durante a diástole ^7^ ( Figura 1 ).

**Figura 2:**
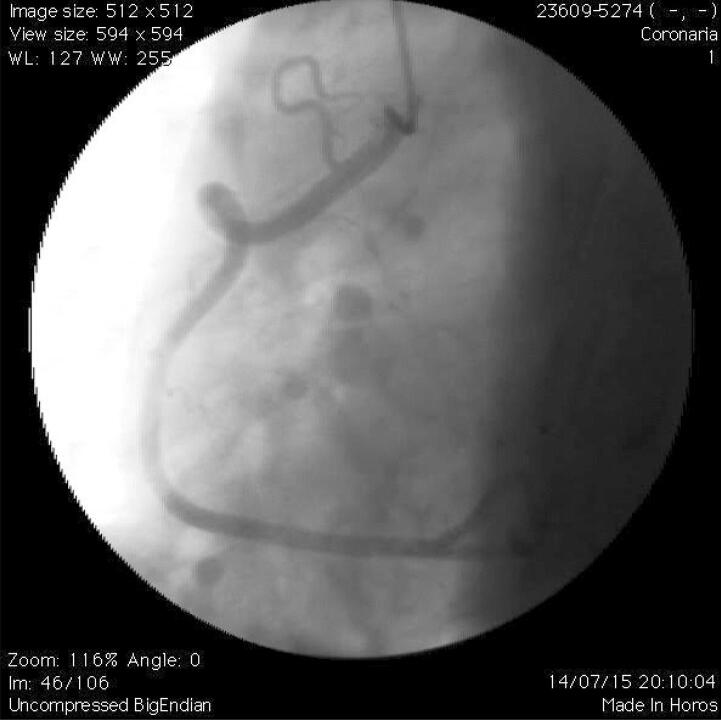
Angiografia coronária mostrando curvatura grave: loop da artéria coronária.

Para a análise dos parâmetros geométricos, foi considerada a definição de TCor grave. Assim, foram medidos o ângulo das curvaturas (ângulo formado pela interseção de duas linhas no ponto do exato onde ocorre a mudança de direção do fluxo sanguíneo – Figura 1 ) e o ângulo mais agudo (quanto mais agudo o ângulo, mais tortuoso é a artéria). Observe as Figuras 2 e 3 mostrando a TCor em diferentes projeções angiográficas.

**Figura 3:**
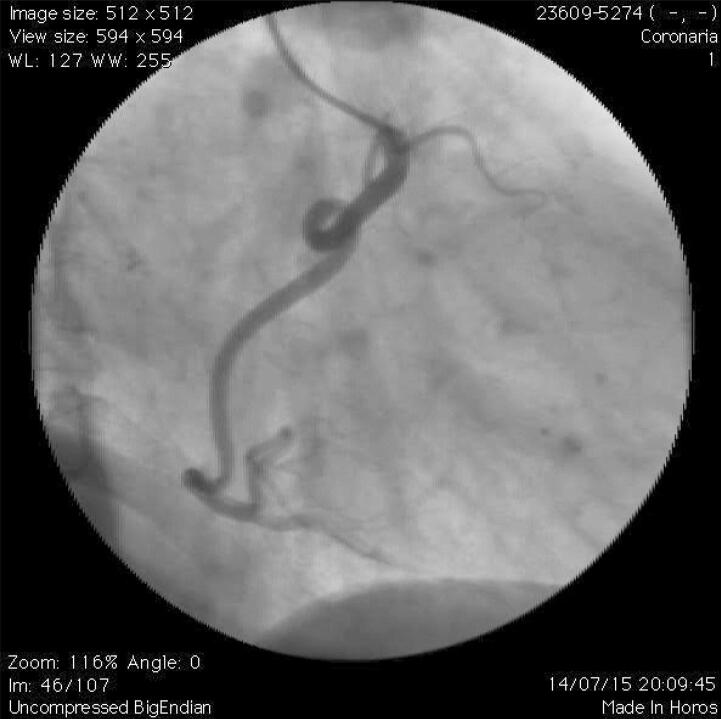
Angiografia coronária mostrando loop da artéria coronária em visão ortogonal à apresentada na Figura 2.

A análise angiográfica foi realizada na diástole e na sístole, nas artérias descendente anterior esquerda (ADAE), circunflexa (ACX), e coronária direita (ACD) (além da artéria descendente posterior no caso de dominância esquerda). As medidas angiográficas da ACX foram realizadas a 30 graus da visão oblíqua anterior esquerda com 30 graus de angulação caudal, e a 30 graus da visão oblíqua anterior direita com 30 graus de angulação caudal. As medidas da ADAE foram realizadas a 30 graus da visão oblíqua anterior direita com 60 graus de angulação cranial, e a 30 graus da visão oblíqua anterior esquerda com 60 graus de angulação cranial. As medidas da artéria coronária direita foram realizadas a 30 graus da visão oblíqua anterior direita e a 30 graus da visão oblíqua anterior esquerda.

A análise das imagens de ICA foi realizada por um observador cego em relação aos resultados da cintilografia miocárdica.

### Avaliação de perfusão miocárdica por imagem

O exame de imagem para perfusão miocárdica (PM) foi realizado para a avaliação fisiológica da presença e local da isquemia miocárdica em todos os pacientes. As imagens foram adquiridas por meio dos aparelhos Millenium MPR (General Electric, Nova Iorque, Estados Unidos) e Infinia Hawk Eye (General Electric, Nova Iorque, Estados Unidos).

As imagens foram interpretadas por médicos da divisão de Medicina Nuclear dos respectivos hospitais, e revisadas por um examinador experiente. Os segmentos com déficits na captura do radiotraçador que se normalizaram nas imagens adquiridas em repouso foram definidas como isquêmicos. Foi usada a segmentação do miocárdio em 17 segmentos, seguindo as diretrizes do comitê de imagens cardíacas da comissão de cardiologia clínica da Associação Americana do Coração ( *Cardiac Imaging Committee of the American Heart Association’s Clinical Cardiology Board* ). ^8^

Os 28 pacientes que se submeteram à cintilografia miocárdica após a ACI foram avaliados de modo cego.

### Análise estatística

As variáveis categóricas foram apresentadas como valores absolutos e porcentagem. As variáveis contínuas com distribuição normal foram apresentadas em média e desvio padrão e as variáveis contínuas sem distribuição normal em mediana e intervalo interquartil. Para avaliar a associação entre variáveis clínicas individuais e variáveis categóricas da TCor, foi usada regressão logística (análise bivariada). As variáveis explanatórias clínicas e cardíacas foram avaliadas de acordo com a presença e a ausência de TCor com o risco relativo (RR) correspondente, seu intervalo de confiança respectivo (IC95%) e o nível descritivo (valor p). Análise de regressão multivariada foi realizada para identificar preditores independentes para o desfecho TCor. As variáveis explanatórias incluídas na regressão multivariada foram as mesas da análise bivariada, por regressão logística. O processo de seleção das variáveis foi o método *stepwise* , com nível de significância de 5%. As diferenças entre os grupos quanto os parâmetros numéricos de angiografia coronária e isquemia foram analisados pelo teste de Mann-Whitney e, para os parâmetros categóricos pelo teste do qui-quadrado (χ2) ou teste exato de Fisher. Realizou-se uma análise prévia para verificar a normalidade das variáveis. Para tanto, realizou-se o teste de Shapiro-Wilk, juntamente com uma análise gráfica dos histogramas. A significância estatística foi definida em 5%. A análise estatística foi realizada usando o programa de estatística SAS®, versão 6.11 (SAS Institute, Inc., Cary, Carolina do Norte).

## Resultados

### Características basais

Características clínicas, angiográficas, e de PM por SPECT (tomografia computadorizada por emissão de fóton único) estão listadas na Tabela 1 . Os pacientes tinham idade média de 58,3 ± 8,8 anos e índice de massa corporal médio de 29 ± 5,2 Kg/m ^2^ , e majoritariamente do sexo feminino (70,2%). A maioria dos pacientes apresentaram angina pectoris classe II ou III (71,9%) de acordo com a classificação da SCC. Os pacientes eram muito sintomáticos, sendo que 56% dos pacientes apresentavam dispneia. O teste de estresse escolhido foi o teste com dipiridamol em 39 pacientes (68%), exercício em 17 pacientes (29,8%) e dobutamina em um paciente (1,7%). A cintilografia miocárdica foi anormal em 37 pacientes (64,9%), com área média de isquemia de 5,9% ± 3,3%. Vinte pacientes apresentaram resultados normais de cintilografia. Os segmentos miocárdicos que apresentaram o maior déficit de perfusão transiente foram aqueles irrigados pela ADAE (43,9%), seguidos da ACX (33,3%) e ACD (22,8%). A ACI mostrou presença de TCor em 28.1% dos pacientes, e a prevalência de tortuosidade foi maior na ADAE e ACX (17,5% cada) e mais baixa na ACD (7%).

**Tabela 1:** Características clínicas, de perfusão miocárdica por tomografia computadorizada por emissão de fóton único (SPECT), e angiográficas dos 57 pacientes incluídos na análise

Características	Valores
**Demográficas**	
Idade (anos), média ± DP	58,3 ±8,8
Sexo feminino	40 (70,2%)
Índice de massa corporal (Kg/m ^2^ ), média ± DP	29 ±5,2
*Clearance* de creatinina (mL/min)	93,6±29,4
**Sintomas presentes**	
Angina CCS I	14 (24,6%)
Angina CCS II	21 (36,8%)
Angina CCS III	20 (31,5%)
Angina CCS IV	2 (3,5%)
Dispneia	32 (56,1%)
**Fatores de risco cardiovascular**	
Tabagismo	8 (14%)
Sedentarismo	47 (82,5%)
Hiperlipidemia	27 (47,4%)
Diabetes	17 (29,8%)
Hipertensão	51 (89,5%)
Fração de ejeção do ventrículo esquerdo (%), média ± DP	66,5 (10,2)
**Medicamentos**	
Betabloqueador ou bloqueador de canal de cálcio	42 (73,7%)
Nitratos ou trimetazidina	34 (59,6%)
Estatinas	30 (52,6%)
Aspirina	48 (84,2%)
**Perfusão miocárdica (SPECT)**	
SPECT anormal	37 (64,9%)
Se anormal, disfunção miocárdica (%), média ± DP	5,9 (3,3)
SPECT anormal no território da ADAE	25 (43,9%)
SPECT anormal no território da ACX	19 (33,3%)
SPECT anormal no território da ACD	13 (22,8%)
**Angiografia coronária invasiva**	
Indivíduos com TCor	16 (28,1%)
TCor na ADAE	10 (17,5%)
TCor na ACX	10 (17,5%)
TCor na ACD	4 (7%)

*CCS: classificação da Canadian Cardiovascular Society, ADAE: artéria descendente anterior esquerda; ACX: artéria circunflexa; ACD: artéria coronária direita; TCor: tortuosidade das artérias coronárias.*

A idade foi o único preditor independente de TCor em nossa amostra (p = 0,042; RR=1,08; CI=1,03-1,17), como pode ser visto na Tabela 2 .

**Tabela 2:** Comparação das características clínicas por resultados angiográficos (presença ou ausência de tortuosidade das artérias coronárias)

Características	Com TCor (n = 16)	Sem TCor (n = 41)	Valor p
Idade (anos)	62,2 ± 7,5	56,8 ± 8,9	0,042
Sexo feminino	13 (81,3%)	27 (65,9%)	0,26
IMC (Kg/m ^2^ )	29,2 ± 5,0	28,9 ± 5,3	0,84
Ácido acetilsalicílico	14 (87,5%)	34 (82,9%)	0,67
Estatina	8 (50,0%)	22 (53,7%)	0,80
Betabloqueador/ bloqueador de canal de cálcio	13 (81,3%)	29 (70,7%)	0,42
Nitrato/ trimetazidina	8 (50,0%)	26 (63,4%)	0,36
Angina CCS I	2 (12,5%)	12 (29,3%)	
Angina CCS II	7 (43,8%)	14 (34,1%)	0,22
Angina CCS III/IV	7 (43,8%)	15 (36,6%)	0,25
Dispneia	9 (56,3%)	23 (56,1%)	0,99
Hipertensão	15 (93,8%)	36 (87,8%)	0,52
Diabetes	5 (31,3%)	12 (29,3%)	0,88
Dislipidemia	6 (37,5%)	21 (51,2%)	0,35
Sedentarismo	13 (81,3%)	34 (82,9%)	0,88
Tabagismo	2 (13%)	6 (14,6%)	0,84

*IMC: índice de massa corporal; TCor: tortuosidade das artérias coronárias.*

### Associação entre isquemia e presença de TCor por vaso e nas amostras de territórios arteriais

Avaliada por vaso (n=171), a associação entra TCor e isquemia foi altamente significativa. A frequência de isquemia em territórios com TCor *versus* territórios sem TCor foi 67% *versus* 28% (p<0,0001). A presença de TCor foi associada com isquemia na ACX (80% vs 21%; p=0,001) e na ACD (75% vs. 19%; p=0,034) mas não na ADAE (50% vs 42%; p=0,46).

### Associação entre isquemia e parâmetros angiográficos por tipo de vaso

A Tabela 3 apresenta o número de casos, mediana, mínimo e máximo, e nível descritivo correspondente (valor p) do teste de Mann-Whitney dos parâmetros da ACI, por ocorrência de isquemia e tipo de artéria.

**Tabela 3:** Isquemia segundo parâmetros angiográficos, por vaso

	Isquemia			Sem isquemia			Valor p

	N	Mediana	Intervalo	N	Mediana	Intervalo	
**ADAE**							
Maior grau de curvatura durante diástole (graus)	25	114	82-135	32	108	78,5-136	0,6
Número de ângulos <90° durante diástole	25	0	0-1	32	0,0	0-1	0,89
Maior grau de curvatura durante sístole (graus)	25	78	59,5-112	32	74,5	61,3-117	0,92
Número de ângulos <90° durante sístole	25	1	0-2,5	32	1	0-3	0,9
**ACX**							
Maior grau de curvatura durante diástole (graus)	19	79	58-109	38	102	74,3-120	0,083
Número de ângulos <90° durante diástole	19	1	0-3	38	0	0-1	0,025
Maior grau de curvatura durante sístole (graus)	19	55	46-96	38	97	52,5-121	0,077
Número de ângulos <90° durante sístole	19	3,0	0-3	38	0	0-2	0,005
**ACD**							
Maior grau de curvatura durante diástole (graus)	13	88	59,5-106,5	44	104	74,8-121	0,16
Número de ângulos <90° durante diástole	13	1	0-1,5	44	0	0-1	0,31
Maior grau de curvatura durante sístole (graus)	13	71	44-93	44	94	57,5-112	0,14
Número de ângulos <90° durante sístole	13	1	0-2	44	0	0-1	0,24

*ADAE: artéria descendente anterior esquerda, ACX: artéria circunflexa; ACD: artéria coronária direita.*

Nesta amostra, não foi observada associação entre isquemia e parâmetros de tortuosidade na ADAE ou na ACD, mas essa associação foi significativa para o número de curvaturas consecutivas com ângulo <90° (p=0,0025) e para o número de curvaturas com ângulo < 90° medidos na sístole (p=0,005) na ACX.

### Associação entre isquemia e parâmetros angiográficos nas amostras de territórios arteriais

A Tabela 4 apresenta o número de casos, mediana, mínimo e máximo, e nível descritivo correspondente (valor p) do teste de Mann-Whitney dos parâmetros da ACI, por ocorrência de isquemia e territórios arteriais (n=171). Houve uma associação significativa entre isquemia e número de curvaturas com ângulos <90° medido na sístole (p = 0,021) nos territórios arteriais.

**Tabela 4:** Parâmetros angiográficos relacionados à presença de isquemia em todas as artérias

Parâmetros angiográficos	Isquemia (n =57)		Sem isquemia (n = 114)		Valor p

	mediana	Intervalo	mediana	Intervalo	
Maior grau de curvatura durante diástole (graus)	92	67-118	105	76-122	0,3
Número de ângulos <90° durante diástole	0	0-2,5	0	0-1	0,1
Maior grau de curvatura durante sístole (graus)	73	48,5-107	85,5	56,5-115	0,074
Número de ângulos <90° durante sístole	1,5	0-3	1	0-2	0,021

## Discussão

Nosso estudo é dedicado a um fenômeno que é cada vez mais reconhecido na prática clínica. Há evidências importantes de que pacientes com isquemia sem obstrução coronária não apresentam um prognóstico benigno, mas, até o momento, não existem diretrizes que orientem a prática clínica. ^6^

Embora a ACI não tenha boa sensibilidade para diagnosticar doenças coronárias funcionais, o exame pode claramente detectar algumas anormalidades, tais como TCor. Até o presente, em nosso conhecimento, não existem estudos que avaliem se a TCor representa outro mecanismo fisiopatológico que leva à isquemia, ou se é um marcador de disfunção microvascular coronariana.

Reconhecer a presença de diferentes mecanismos de isquemia nesses pacientes seria importante para se desenvolver a medicina estratificada, uma nova abordagem terapêutica para os pacientes. No ensaio CorMicA, ^9 , 10^ assim como em muitos outros estudos, as mulheres eram predominantes e apresentaram um fenótipo diferente de DAC na angiografia coronária em comparação aos homens, devido a um menor número de obstruções coronárias e reserva de fluxo coronário reduzido, achados associados com eventos cardiovasculares maiores tais como morte cardiovascular e hospitalização por infarto do miocárdio e insuficiência cardíaca. ^11^ Li et al. ^12^ demonstraram que a TCor está positivamente relacionada com hipertensão e sexo feminino, mas negativamente associado com DAC.

El. Tahlawi et al. ^13^ descreveram que a TCor está associada com aterosclerose subclínica e com escore de cálcio coronariano aumentado mesmo na ausência de lesão obstrutiva significativa. Outro estudo foi conduzido mostrando a relação entre espessura da camada íntima-média carótida e presença de TCor, e na presença de tortuosidade associada na artéria retinal, sugerindo, assim, uma associação com a forma subclínica de aterosclerose. ^14^

A alta prevalência de indivíduos do sexo feminino, idade avançada, e hipertensão é observada em pacientes com Tcor e em pacientes com disfunção microvascular coronariana. ^6 , 15 - 17^ Podemos comparar nossos resultados com aqueles apresentados em dois outros estudos em que se utilizou a mesma definição de Tcor e foi demonstrada uma correlação entre Tcor e isquemia. Gaibazzi et al. ^16^ encontraram, em um subgrupo de 34 pacientes com as mesas características (angina aos esforços e teste provocativo positivo), a prevalência de 27,3% (n=9). Yang et al. ^12^ observaram uma prevalência de 37,5% em uma amostra de 48 pacientes. Gaibazzi et al. ^18^ e Yang et al. ^12^ não encontraram nenhum fator de risco cardiovascular relacionado à presença de TCor, como em nosso estudo.

Observamos uma relação significativa, já descrita na literatura, de TCOr com idade avançada ^16 , 19 , 20^ (p=0,042). Assim, a TCor parece ser o resultado final de mudanças estruturais e funcionais do coração e talvez represente um mecanismo de adaptação que permita que o coração mude dinamicamente seu tamanho e sua função. ^21^ Isso pode depender da hipertrofia ventricular esquerda e relaxamento deficiente concomitante, o que parece ser mais comum em idosos. Uma possível explicação é que a hipertrofia possa afetar o padrão geodésico das artérias coronárias provavelmente devido a fatores angiogenéticos, o que pode ser mediado pelo fluxo sanguíneo, estresse da parede, e fatores de crescimento. ^21^

Diferentemente de outros estudos, nós analisamos a relação entre TCOr e isquemia por vaso e território correspondente. Em nossa amostra, TCor esteve presente na ACX e na ADAE em 10 pacientes (17,5%) e na ACD em quatro pacientes (7%). Houve uma relação significativa entre TCor e isquemia na ACX e na ACD, o que não foi observado no território da ADAE. Achados angiográficos anormais de TCor foram mais evidentes na ACX (número de curvaturas tanto na sístole como na diástole, e menor ângulo de curvatura medido tanto na sístole como na diástole), o que pode explicar a maior frequência de isquemia neste território.

Nosso estudo é o primeiro a demonstrar que um parâmetro específico de tortuosidade do vaso está relacionado com a presença de isquemia miocárdica. O maior número de curvaturas detectadas durante a sístole na angiografia esteve relacionado com isquemia miocárdica em pacientes sem obstruções coronárias. Estudos investigando mudanças específicas na geometria coronária e sua correlação com isquemia do miocárdio são escassos. Hassan et al. ^22^ criaram um índice de gravidade da tortuosidade e observaram que esse foi um forte preditor de dor anginal entre os pacientes com artérias coronárias normais, apesar de um teste de estresse positivo. Porém, os autores não avaliaram a presença de isquemia nos territórios coronarianos como nós fizemos.

A relação da isquemia com TCor foi diferente entre os territórios coronarianos. Não se observou associação entre TCor e isquemia na ADAE. Yokota et al. ^23^ estudaram um grupo de pacientes com SPECT normal e sintomas persistentes usando a reserva de fluxo fracionada (FFR, *functional flow reserve* ). Neste estudo, os autores encontraram que a FFR foi significativamente mais anormal na ADAE, demonstrando que a diferente quantidade de miocárdio nos territórios coronarianos pode criar interações heterogêneas com anatomia coronária e isquemia. ^23^ A massa de miocárdio subtendida por uma lesão é um importante fator preditor de uma FFR < 0,80 como demonstrado por Yoon et al. ^24^ Novos métodos de estimar o comprometimento hemodinâmico no fluxo coronário, tais como o *contrast-flow quantitative flow ratio* ( *cQFR* ), demonstraram que o mesmo tipo de discrepâncias em comparação às medidas de SPECT do miocárdio. ^25^ Podemos especular que as diferenças nos territórios irrigados pelas artérias coronárias possam explicar parte de nossos resultados, uma vez que o aumento na massa miocárdica pode recrutar mais vasos colaterais na microcirculação.

Um resultado importante é a necessidade de uma definição mais precisa e uniformemente aceita de TCor para a padronização de novos estudos. ^26^ É importante a adoção de mais de uma variável angiográfica além da gravidade e do número dos ângulos, bem como as medidas dinâmicas em ambas as fases do ciclo cardíaco (sístole e diástole). Isso se torna ainda mais importante quando observamos que a angiografia coronária realiza somente medidas bidimensionais de uma estrutura altamente dinâmica com três dimensões. ^27^ Estudos sobre fluidodinâmica computacional chamam a atenção à importância das medidas realizadas por equações matemáticas complexas que explicariam melhor como ocorre a distribuição da pressão ao longo da circulação e do fluxo coronários. ^28 - 34^

## Limitações

**Figura 4:**
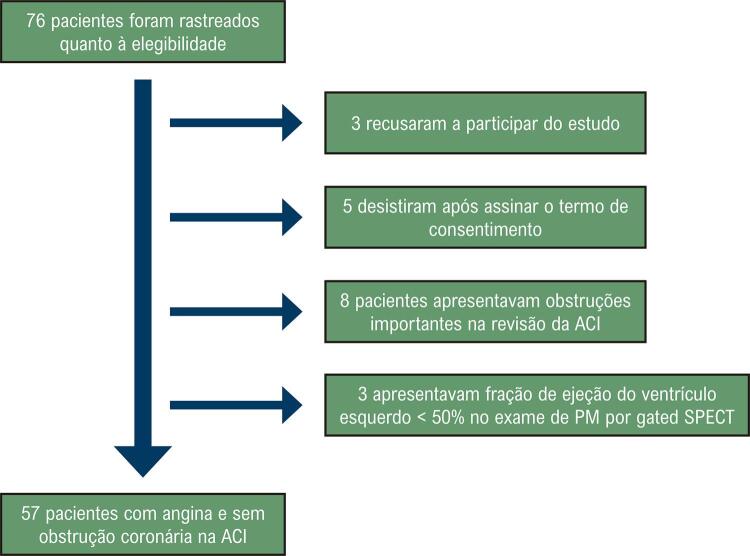
Fluxograma da pesquisa; PM: perfusão miocárdica; gated SPECT: tomografia computadorizada por emissão de fóton único de sincronização cardíaca; ACI: angiografia coronária invasiva.

Nosso estudo tem algumas limitações. A primeira limitação é o pequeno tamanho amostral e sua natureza retrospectiva ( Figura 4 ). Nós não usamos informação angiográfica para estabelecer um índice de tortuosidade, o que pode ser proposto por estudos futuros. Não realizamos exames para avaliar a função coronariana, por esses não serem utilizados rotineiramente na prática clínica.

## Conclusões

A TCor está associada com isquemia miocárdica em casos selecionados. O número de curvaturas avaliado na sístole na angiografia coronária está associado a um risco aumentado de isquemia miocárdica. É necessária uma análise individualizada da anatomia da artéria coronária e seu território correspondente antes de se considerar um resultado falso-positivo na cintilografia miocárdica em pacientes com TCor.
