# Supplementary material for: Tortuosidade das Artérias Coronárias como um Novo Fenótipo para Isquemia sem Doença Arterial Coronariana
Source: Arq Bras Cardiol. 2022 Sep 15;119(6):883–90. [Article in Portuguese] doi: 10.36660/abc.20210787 (PMC9814816; doi:10.36660/abc.20210787)
Supplement: Translation

# Translation: Coronary Tortuosity as a New Phenotype for Ischemia without Coronary Artery Disease

**Authors:** André Estrada, André Silveira Sousa, Claudio Tinoco Mesquita, Humberto Villacorta

**Affiliations:** 1 Brazil Universidade Federal Fluminense Hospital Universitário Antônio Pedro,Niterói, RJ – Brazil; 2 Brazil Hospital Pró-Cardíaco, Rio de Janeiro, RJ – Brazil; 3 Brazil Universidade Federal Fluminense Faculdade de Medicina – Departamento de Radiologia, Niterói, RJ – Brazil; 4 Brazil Universidade Federal Fluminense – Cardiologia, Niterói, RJ – Brazil

**Keywords:** Coronary Vessels, Ischemia, Myocardial Ischemia

## Abstract

**Background:**

Coronary arteries tend to be more tortuous than other arteries and follow the repeated flexion and relaxation movements that occur during the cardiac cycle. Coronary tortuosity (CorT) leads to changes in coronary flow with a reduction in distal perfusion pressure, which could cause myocardial ischemia.

**Objective:**

To assess the association between CorT and myocardial ischemia.

**Methods:**

Between January 2015 and December 2017, 57 patients with angina and nonobstructive coronary artery disease detected by invasive coronary angiography (ICA) were retrospectively enrolled. Angiographic variables were analyzed to assess the presence and degree of tortuosity and correlated with their respective vascular territories on stress myocardial perfusion imaging (MPI). CorT was defined as coronary arteries with three or more bend angles ≤90°, measured during diastole. Statistical significance was determined at the 5% level.

**Results:**

A total of 17 men and 40 women were enrolled (mean age 58.3 years). CorT was observed in 16 patients (28%) and in 24 of 171 arteries. There was a significant association between CorT and ischemia when analyzed per artery (p<0.0001). The angiographic factor most associated with ischemia was the number of bend angles in an epicardial artery measured at systole (p=0.021).

**Conclusion:**

This study showed an association of CorT and myocardial ischemia in patients with unobstructed coronary arteries and angina. An increased number of coronary bend angles measured by angiography during systole was related to ischemia.

## Introduction

Ischemic heart disease is the leading cause of death in the developed world and limits patient quality of life in physical, social, financial and health aspects.^1^ The recently published European Society of Cardiology Guidelines for the diagnosis and management of chronic coronary syndromes (CCSs) describe clinical scenarios in patients with a suspected or established CCS.^2^ The clinical profile of angina with nonobstructive epicardial vessels has been increasingly recognized and associated with obesity, glucose intolerance and longer life expectancy.^3^ Studies have suggested that up to 55% of patients referred for coronary angiography, even with typical symptoms, have no obstructions, and up to 40% of patients with normal or near-normal arteries (without obstructive lesions) on coronary angiography have ischemia, as demonstrated by stress tests.^4^ Patients with angina pectoris who do not have significant coronary obstruction are still at increased risk for major cardiovascular events such as cardiovascular death, acute myocardial infarction, stroke, heart failure, and all-cause mortality.^5^ These patients are also at a higher risk of heart failure with preserved ejection fraction.^6^

A possible mechanism related to ischemia in nonobstructive disease is coronary tortuosity (CorT). Reduction in distal perfusion pressure and coronary flow, leading to the appearance of myocardial ischemia can be observed in some cases of CorT. There are two causes for this pressure reduction: friction due to shear stress and the centrifugal effect within curves.^7^ This association has been poorly addressed in the literature. The primary aim of this study was to assess the correlation between CorT and myocardial ischemia in patients without coronary obstruction, and the secondary aim was to verify the geometrical characteristics of each coronary vessel that could be correlated with ischemia.

## Materials and methods

### Patient selection

This was a retrospective study conducted at two medical centers. The study was approved by the institutional review board. All patients provided written informed consent before participating in the study.

A total of 57 subjects were included in the final analysis. We selected patients who underwent provocative tests with ischemic alterations and coronary angiography without obstructions. Of these patients, 28 patients had undergone a positive exercise test but had not undergone myocardial scintigraphy, which was then performed prospectively. The maximum interval between myocardial scintigraphy and coronary angiography was one year, regardless of the order in which they were performed.

Patients were enrolled if they were ≥18 years of age, had clinical complaints of angina pectoris, and had undergone invasive coronary angiography (ICA) that revealed no obstructive lesions (a nonobstructive lesion was defined as a lack of obstruction or an obstruction of less than 30%). Patients were excluded from the study if any of the following conditions were observed: heart failure, pulmonary hypertension, congenital disease, valvular heart disease, previous myocardial revascularization (surgical or percutaneous), hypertrophic cardiomyopathy, myocarditis, myocardial bridge, congenital anomalies of coronary origin (distribution and course), arteriovenous fistulas and coronary artery-left ventricular microfistulas, catheter-induced coronary spasm, anemia (hemoglobin <10 g/d/L), complete left bundle branch block or definitive pacemaker.

### Clinical Data

Medical records were reviewed, and interviews were conducted with the patients. The functional class of the angina was verified according to the Canadian Cardiology Society^2^ criteria, in addition to associated symptoms of dyspnea, history of comorbidities such as hypertension, diabetes mellitus, dyslipidemia, history of smoking and physical inactivity, and the available complementary exams.

### Invasive coronary angiography

The ICA was used to rule out the presence of coronary obstructions and myocardial bridges and to assess the presence of CorT and its grading. Quantitative coronary angiographic analysis was performed using standard techniques. Severe CorT was defined as the presence of at least three consecutive curvatures with a curvature angle of less than 90 degrees of an epicardial coronary artery greater than 2 mm during diastole^7^ ( Figure 1 ).

**Figure 1:** Coronary angiography demonstrating how the bend angle is measured.

For analysis of geometric parameters, the definition of severe CorT was considered. Thus, measurements were made of the bend angle (the angle formed by the intersection of the two lines at the exact point where the change in blood flow direction occurs- Figure 1 ) and of the most severe bend angle (the more acute the angle, the more tortuous the artery is). Note Figures 2 and 3 that show CorT in different angiographic projections.

**Figure 2:** Coronary angiography showing severe curvature: coronary loop.

**Figure 3:** Coronary angiography showing coronary loop in an orthogonal view to that shown in Figure 2.

Angiographic analysis was performed in diastole and in systole, in the left anterior descending (LAD), left circumflex (LCX), and right coronary (RCA) arteries (also of the left posterior descending artery in case of left dominance). The angiographic measurements of the LCX were made at the 30-degree left anterior oblique view with 30 degrees of caudal angulation, and at the 30-degree right anterior oblique view with 30 degrees of caudal angulation. Measurements of the LDA were made on the 30-degree right oblique anterior view with 60-degree cranial, and on the 30-degree anterior oblique left view with 60-degree cranial. Measurements of the right coronary artery were made on the 30-degree right anterior oblique view and on the 30-degree left anterior oblique view.

The analysis of the ICA images was performed by one observer blind with respect to the myocardial scintigraphy results.

### Myocardial perfusion imaging

Myocardial perfusion imaging (MPI) was performed for the physiological evaluation of the presence and location of myocardial ischemia in all patients. Images were acquired on a Millenium MPR (General Electric, New York, United States) and an Infinia Hawk Eye (General Electric, New York, United States).

Images were interpreted by physicians of the Nuclear Medicine sectors of the respective hospitals and reviewed by an experienced examiner. Segments with deficits in the capture of radiotracer that normalized in the images acquired at rest were defined as ischemic. The 17-segment myocardial segmentation model was used, following the guidelines of the Cardiac Imaging Committee of the American Heart Association’s Clinical Cardiology Board.^8^

The 28 patients who underwent myocardial scintigraphy after ICA were analyzed in a blinded manner.

### Statistical analysis

Categorical variables were presented as absolute value and percentage. Continuous variables with normal distribution were expressed as mean and standard deviation and continuous variables with non-normal distribution as median and interquartile range. To evaluate the association between individual clinical variables and cardiological variables of CorT, binary logistic regression (bivariate analysis) was used. The clinical and cardiac explanatory variables were analyzed according to the presence or absence of CorT with the corresponding relative risk (RR), its respective confidence interval (95% CI) and the descriptive level (p value). Multivariable regression analysis was performed to identify independent predictors for the CorT outcome. The explanatory variables included in the multivariate regression were the same as those in the bivariate analysis, by logistic regression. The selection process of the variables was the stepwise forward method at the 5% level. The differences between the groups regarding numerical coronary angiography parameters and ischemia were analyzed by the Mann-Whitney U test and, for categorical parameters, by the chi-squared test (χ2) or Fisher’s exact test. A prior analysis was performed to verify the normality of the variables. For this, the Shapiro-Wilk test was used together with a graphical analysis of the histograms. Statistical significance was determined at the 5% level. Statistical analysis was performed using SAS® System statistical software, version 6.11 (SAS Institute, Inc., Cary, North Carolina).

## Results

### Baseline characteristics

Clinical, myocardial perfusion (single-photon emission computer-assisted tomography, SPECT) and angiographic characteristics of the study participants are described in Table 1 . The patients had a mean age of 58.3 ± 8.8 years and a mean body mass index of 29 ± 5.2 Kg/m^2^ and was predominantly female (70.2%). Most patients had angina pectoris class II or III (71.9%) according to the Canadian Society of Cardiology classification. Patients were very symptomatic, with dyspnea being present in 56% of patients. Dipyridamole was the selected stress test in 39 patients (68%), exercise in 17 patients (29.8%) and dobutamine in one patient (1.7%). Myocardial scintigraphy was abnormal in 37 patients (64.9%) with a mean ischemic area of 5.9% ± 3.3%. Twenty patients had normal scintigraphy. The myocardial segments that showed the greatest transient perfusion deficit were those that were supplied by the LAD (43.9%), followed by the LCX (33.3%) and the right coronary artery (22.8%). ICA showed CorT in 28.1% of the patients, and the prevalence of tortuosity was higher in the LAD and LCX (17.5% each) and lower in the RCA (7%).

**Table 1:** Clinical, myocardial perfusion SPECT, and coronary angiographic characteristics of 57 patients included in the analysis

Characteristic	Value
**Demographics**	
Age (years), mean ± SD	58.3 ±8.8
Female sex	40 (70.2%)
Body mass index (Kg/m2), mean ± SD	29 ±5.2
Creatinine Clearance (mL/min)*	93.6±29.4
**Presenting symptoms**	
Angina CCS I	14 (24.6%)
Angina CCS II	21 (36.8%)
Angina CCS III	20 (31.5%)
Angina CCS IV	2 (3.5%)
Dyspnea	32 (56.1%)
**Cardiovascular risk factors**	
Smoking	8 (14%)
Sedentarism	47 (82.5%)
Hyperlipidemia	27 (47.4%)
Diabetes	17 (29.8%)
Hypertension	51 (89.5%)
Left ventricular ejection fraction (%), mean ± SD	66.5 (10.2)
**Medication**	
Beta-blocker or calcium channel blockers	42 (73.7%)
Nitrates or trimetazidine	34 (59.6%)
Statins	30 (52.6%)
Aspirin	48 (84.2%)
**Myocardial perfusion SPECT**	
Abnormal SPECT	37 (64.9%)
If abnormal, myocardial impairment (%), mean ± SD	5.9 (3.3)
Abnormal SPECT in LAD territory	25 (43.9%)
Abnormal SPECT in LCX territory	19 (33.3%)
Abnormal SPECT in RCA territory	13 (22.8%)
**Invasive Coronary Angiography**	
Subjects with CorT	16 (28,1%)
LAD CorT	10 (17,5%)
LCX CorT	10 (17,5%)
RCA CorT	4 (7%)

*Cockcroft-Gault equation; SD: standard deviation. SPECT: single-photon emission computer-assisted tomography; CCS: Canadian Cardiovascular Society classification, LAD: left anterior descending artery; LCX: left circumflex artery; RCA: right coronary artery; CorT: coronary tortuosity.

Age was the only significant independent predictor for CorT in our sample (p = 0.042; RR=1.08; CI=1.03-1.17) as we can see in Table 2 .

**Table 2:** Comparison of clinical characteristics by angiographic results (presence or absence of coronary tortuosity)

Characteristics	CorT (n = 16)	No CorT (n = 41)	p value
Age (years)	62.2 ± 7.5	56.8 ± 8.9	0.042
Female	13 (81.3%)	27 (65.9%)	0.26
BMI (kg/m^2^)	29.2 ± 5.0	28.9 ± 5.3	0.84
Acetylsalicylic acid	14 (87.5%)	34 (82.9%)	0.67
Statin	8 (50.0%)	22 (53.7%)	0.80
Beta-blocker/ calcium channel blocker	13 (81.3%)	29 (70.7%)	0.42
Nitrate/ trimetazidine	8 (50.0%)	26 (63.4%)	0.36
Angina CCS I	2 (12.5%)	12 (29.3%)	
Angina CCS II	7 (43.8%)	14 (34.1%)	0.22
Angina CCS III/IV	7 (43.8%)	15 (36.6%)	0.25
Dyspnea	9 (56.3%)	23 (56.1%)	0.99
Hypertension	15 (93.8%)	36 (87.8%)	0.52
Diabetes	5 (31.3%)	12 (29.3%)	0.88
Dyslipidemia	6 (37.5%)	21 (51.2%)	0.35
Sedentarism	13 (81.3%)	34 (82.9%)	0.88
Smoker	2 (13%)	6 (14.6%)	0.84

*BMI: body mass index; CorT: coronary tortuosity.*

### Association between ischemia and the presence of CorT per vessel and in the sampled vessel territories

Evaluated per vessel (n = 171), the association between CorT and ischemia was highly significant. The frequency of ischemia in territories with CorT *versus* territories without CorT was 67% *versus* 28% (p<0.0001). The presence of CorT was associated with ischemia in the LCX (80% vs 21%; p=0.001) and the RCA (75% vs 19%; p=0.034) but not in the LAD (50% vs 42%; p=0.46).

### Association between ischemia and angiographic parameters by vessel type

Table 3 provides the number of cases, median, minimum and maximum and corresponding descriptive level (p value) of the Mann-Whitney U test of the coronary angiography parameters, by occurrence of ischemia and artery type.

**Table 3:** Ischemia according to angiographic parameters per vessel

	Ischemia			No Ischemia			p value

	N	Median	Range	N	Median	Range	
**LAD**							
most severe bend angle during diastole (degrees)	25	114	82-135	32	108	78.5-136	0.6
number of bend angles <90° during diastole	25	0	0-1	32	0.0	0-1	0.89
most severe bend angle during systole (degrees)	25	78	59.5-112	32	74.5	61.3-117	0.92
number of bend angles <90° during systole	25	1	0-2.5	32	1	0-3	0.9
**LCX**							
most severe bend angle during diastole (degrees)	19	79	58-109	38	102	74.3-120	0.083
number of bend angles <90° during diastole	19	1	0-3	38	0	0-1	0.025
most severe bend angle during systole (degrees)	19	55	46-96	38	97	52.5-121	0.077
systole number of bend angles <90° during systole	19	3.0	0-3	38	0	0-2	0.005
**RCA**							
most severe bend angle during diastole (degrees)	13	88	59.5-106.5	44	104	74.8-121	0.16
number of bend angles <90° during diastole	13	1	0-1.5	44	0	0-1	0.31
most severe bend angle during systole (degrees)	13	71	44-93	44	94	57.5-112	0.14
number of bend angles <90° during systole	13	1	0-2	44	0	0-1	0.24


*LAD: left anterior descending artery; LCX: left circumflex artery; RCA: right coronary artery.*

In this sample, no association was observed between ischemia and tortuosity parameters in the LAD or RCA, but this association was significant for the number of consecutive bend angles <90° (p = 0.025) and for the number of bend angles <90° measured at systole (p = 0.005) in the LCX.

### Association between ischemia and angiographic parameters in the sampled vessel territories

Table 4 provides the number of cases, median, minimum and maximum and the corresponding descriptive level (p value) of the Mann-Whitney U test of coronary angiography parameters by occurrence of ischemia and arterial territories (n = 171). There was a significant association between ischemia and the number of bend angles <90° measured at systole (p = 0.021) in the sampled vessel territories.

**Table 4:** Angiographic parameters related to the presence of ischemia in all arteries

Angiographic parameters	Ischemia (n =57)		No Ischemia (n = 114)		p value

	median	Range	median	range	
most severe bend angle during diastole (degrees)	92	67-118	105	76-122	0.3
number of bend angles <90° during diastole	0	0-2.5	0	0-1	0.1
most severe bend angle during systole (degrees)	73	48.5-107	85.5	56.5-115	0.074
number of bend angles <90° during systole	1.5	0-3	1	0-2	0.021

## Discussion

Our study is dedicated to a phenomenon that has been increasingly recognized in clinical practice. There is considerable evidence that patients with ischemia without coronary obstruction do not have a benign prognosis, but so far, there are no guidelines to guide clinical practice.^6^

ICA lacks the sensitivity to diagnose functional coronary disorders, but can clearly detect some abnormalities, such as CorT. Until now, to our knowledge, there are no studies that assess whether CorT may represent another pathophysiological mechanism that leads to ischemia or be a marker of coronary microvascular dysfunction (CMD).

Recognizing the presence of different mechanisms of ischemia in these patients may be important for performing stratified medicine, a new treatment approach for patients. In the CorMicA trial,^9 , 10^ as in many other studies, women were predominant and presented with a different phenotype of CAD from men on coronary angiography because of a smaller number of coronary obstructions and decreased coronary flow reserve, findings that are associated with major cardiovascular events such as cardiovascular death and hospitalization for myocardial infarction and heart failure.^11^ Li et al.^12^ demonstrated that CorT is positively related to hypertension and female gender, but negatively linked with CAD.

El Tahlawi et al.^13^ described that CorT is associated with subclinical atherosclerosis and increased coronary artery calcium score even in the absence of significant obstructive lesion. Another study was carried out showing the relationship between carotid intima-media thickness and the presence of CorT, and also in the presence of associated tortuosity in the retinal artery, thus suggesting an association with the subclinical form of atherosclerosis.^14^

Increased prevalence of females, advanced age, and hypertension is observed in patients with CorT and in patients with coronary microvascular dysfunction.^6 , 15 - 17^ We can compare our findings with those reported in two other studies that used the same definition of CorT and demonstrated a correlation between CorT and ischemia. Gaibazzi et al.^16^ found in a subgroup of 34 patients with the same characteristics (anginal pain on exertion and positive provocative test) the prevalence of 27.3% of CorT (n=9). Yang et al.^12^ observed a 37.5% prevalence in a sample of 48 patients. Gaibazzi et al.^18^ and Yang et al.^12^ did not find any cardiovascular risk factors related to the presence of CorT, as in our study.

We observed a significant relationship, already described in the literature, of CorT with advanced age^16 , 19 , 20^ (p = 0.042). In this sense, CorT seems to be the end result of structural and functional changes of the heart and perhaps represents an adaptation mechanism that allows the heart to dynamically modify its size and function.^21^ It may be dependent on left ventricular hypertrophy and concomitant impaired relaxation, which have been found to be more common in elderly people. One probable explanation is that hypertrophy might affect the geodesic pattern of coronary arteries plausibly due to angiogenetic factors, which may be mediated by blood flow, wall stress, and growth factors.^21^

Unlike in other studies, we analyzed the relationship between CorT and ischemia by vessel and corresponding territory. In our sample, CorT was present in the LCX and LDA in 10 patients (17.5%) and in the RCA in four patients (7%). The relationship between CorT and ischemia in the LCX and RCA was significant, but this relationship was not found in the LAD territory. Abnormal angiographic findings of CorT were more evident in the LCX (number of bend angles measured in both systole and diastole and smaller bend angle measured in systole and diastole), which may explain the greatest frequency of ischemia in this territory.

Our study is the first to demonstrate that a specific parameter of vessel tortuosity is related to the presence of myocardial ischemia. The highest number of acute bend angles detected during systole in angiography was related to myocardial ischemia in patients without coronary obstructions. Studies investigating specific changes in coronary geometry and their correlation with myocardial ischemia are missing. Hassan et al.^22^ created a tortuosity severity index and found that it was a strong predictor of anginal pain among patients with normal coronary arteries, despite a positive stress study, but they did not evaluate the presence of ischemia in the coronary territories, as we did.

The relation of ischemia with CorT was different among the coronary territories. The LAD did not demonstrate an association of CorT with ischemia. Yokota et al.^23^ studied a group of patients with normal single-photon emission computer-assisted tomography (SPECT) and persistent symptoms using fractional flow reserve (FFR). In this study, they found that the FFR was significantly more abnormal in the LAD, demonstrating that the different amount of myocardium in the coronary territories may create heterogeneous interactions with coronary anatomy and ischemia.^23^ Myocardial mass subtended by a lesion is an important factor predicting an FFR < 0.80, as demonstrated by Yoon et al.^24^ New methods of estimating hemodynamic compromise in coronary flow, such as the contrast-flow quantitative flow ratio (cQFR), demonstrated the same type of discrepancies compared with myocardial SPECT measurements.^25^ We can speculate that the differences in territories supplied by the coronary arteries may explain part of our results because the increased myocardial mass may recruit more collateral vessels in the microcirculation.

One important finding is the need for a more accurate and uniformly accepted definition of CorT to standardize new studies.^26^ The adoption of more than one angiographic variable beyond the severity of bending angles and the number of bend angles, as well as dynamic measurements in both phases of the cardiac cycle (systole and diastole), is important. This becomes even more significant when we note that coronary angiography makes only two-dimensional measurements of a highly dynamic structure that exists in three dimensions.^27^ Studies on computational fluid dynamics draw attention to the importance of measurements made by complex mathematical equations that would best explain how pressure distribution occurs along the coronary circulation and the flow within it.^28 - 34^

## Limitations

Our study has some limitations. The first limitation is the small sample size of the study and its retrospective nature ( Figure 4 ). We did not use angiographic information to establish a tortuosity index, which may be proposed in future studies. We did not perform coronary function testing, as it is not routinely used in clinical practice.

**Figure 4:** Research flowchart; GATED-SPECT: cardiac-gated single-photon emission computer-assisted tomography; MPI: myocardial perfusion imaging; ICA: invasive coronary angiography.

## Conclusions

CorT is associated with myocardial ischemia in selected cases. The number of bend angles assessed by systole in coronary angiography is linked to an increased risk of myocardial ischemia. An individualized analysis of the coronary artery anatomy and its corresponding territory is needed before considering a false-positive myocardial scintigraphy finding in patients with CorT.
